# Report of a Case of Creutzfeldt-Jakob Disease With an Unusual Clinical Presentation

**DOI:** 10.3389/fnbeh.2020.00055

**Published:** 2020-04-09

**Authors:** Elena Prodi, Stefania Rossi, Ilaria Bertaina, Emanuele Pravatà, Leonardo Sacco

**Affiliations:** ^1^Department of Neuroradiology, Neurocenter of Southern Switzerland, Lugano, Switzerland; ^2^Department of Neurology, Neuropsychology and Behavioral Neurology Research Unit, Neurocenter of Southern Switzerland, Lugano, Switzerland; ^3^Department of Neurology, Neurocenter of Southern Switzerland, Lugano, Switzerland

**Keywords:** Creutzfeldt-Jakob disease, sporadic, MRI, PET, diagnostic criteria, RT-QuIC

## Abstract

We describe the clinical features, neuropsychological tests, laboratory, electroencephalography (EEG), magnetic resonance imaging (MRI) and positron emission tomography (PET) findings of a 59-year-old woman who presented to our Centre for cognitive impairment since few months, with language disturbances, particularly anomia, dyscalculia, and memory loss. The clinical and neuropsychological features were non-specific and overlapping with those of other rapidly progressing neurodegenerative disorders. However, brain MRI played a pivotal role in the diagnosis, showing cortical diffusion restriction, particularly in the parietal lobes and posterior cingulum, with sparing of the perirolandic cortex, typical of Creutzfeldt-Jakob disease (CJD). Brain MRI abnormalities were visible since the first evaluation and remained stable at 2 and 6 weeks follow up. Basal ganglia and thalami were never involved. PET showed left lateralized reduced glucose metabolism, with partial overlap with MRI signal abnormalities. Despite MRI were strongly indicative of CJD, clinical, laboratory and EEG findings did not fulfill the diagnostic criteria for CJD which applied at the time of clinical assessment. Indeed, neither myoclonus, visual or cerebellar signs or akinetic mutism were present. Also, the characteristic periodic sharp wave complexes were absent at baseline EEG, and the CSF assay for 14–3–3 was negative. We, therefore, performed a real-time quaking-induced conversion (RT-QuIC) assay on a frozen sample of corticospinal fluid (CSF), which showed a positive result. RT-QuIC is a prion protein conversion assay that has shown high diagnostic sensitivity and specificity for the diagnosis of CJD. RT-QuIC has been recently incorporated in the National CJD Research and Surveillance Unit and Center for Disease Control and Prevention (CDC) diagnostic criteria for CJD. The fatal evolution of the disease brought the patient to death 13 months after symptoms onset. Pathology proved the diagnosis of sporadic CJD, subtype MM/MV 2C.

## Introduction

We describe a case of rapidly progressive dementia (RPD) that started with impairment of language skills and discuss the neuropsychological differential diagnosis. We outline the role of new diagnostic criteria for the diagnosis of Creutzfeldt-Jakob disease (CJD) that allow *in vivo* diagnosis of the disease even in cases with atypical clinical and electroencephalography (EEG) presentation, such as in this case.

## Case Presentation

We report of a 59-year-old woman who developed, since December 2015, language abnormalities characterized by word retrieval difficulties and impairment in sentence production. These changes were reported to interfere with her job activity, with raising work-related stress and anxiety. In the following 6 months, she developed difficulties in calculation abilities, impairment of time perception and short-term memory loss. The social entourage had perceived that something was wrong, especially since spring 2016, when she was referred to a local neurologist and a mini-mental state examination (MMSE) was performed with a normal score (30/30). After 9 months from symptoms onset, she was considered unable to work. She was referred to our neurological outpatient service and hospitalized. Neurological examination in December 2016 showed moderate aphasia with some difficulties in the denomination. No pathological abnormalities were found in the neurologic exam of cranial nerves, strength, tone, reflexes, coordination, sensory function, and gait. A neuropsychological evaluation was conducted during the hospitalization with an initial informal discussion followed by a detailed neuropsychological examination using a battery of tasks designed to evaluate global cognitive functioning (Mini-Mental Status Examination, MMSE), language (Boston Naming test-short version, Phonemic and Semantic Fluency test), short term and working memory (forwards and backward Digit and Corsi span), anterograde verbal episodic memory (Story Recall test), anterograde visuospatial memory (Rey-Osterrieth Complex figure test Recall), attention and processing speed (Trail Making Test), executive function [Frontal Assessment battery (FAB)] and visuospatial skills (Rey-Osterrieth Complex figure test Copy). A detailed language assessment was also conducted through the Neuropsychology Exam for Aphasia (ENPA). At the time of the evaluation, the patient was alert and oriented in all domains, engaged in the examination but mildly anxious. She exhibited partial insight about her declining abilities, claiming that these were due to work-related stress. We perceived a lack of emotional insight and concern over her status. In the spontaneous speech, she showed difficulty in language production, with impaired word-retrieval and use of passe-partout words. There was no evidence of a primary impairment of comprehension. The language was characterized by a lack of focus, tangential and ambiguous speech. The neuropsychological evaluation outlined impairment across multiple domains (Table 1). She scored 23/30 at MMSE, showing a rapid deterioration compared to the test performed 6 months before, in June. Her performance was particularly poor in short and long verbal memory tasks. She was also impaired in the executive domain, with difficulty accessing mental lexicon and carrying out mentally effortful tasks such as calculation, mental manipulation, task-set inhibition and cognitive flexibility. Visuospatial long-term memory was less affected than verbal memory. Other measured abilities appeared to be preserved, including graphomotor skills and visuospatial abilities. The investigation of speech and language functions (Table 2) showed prominent abnormalities in two specific aspects of language processing: verbal fluency (Letter and Category subtasks) and spontaneous speech (Spoken Picture Description). Connected speech was compromised due to frequent pauses for word retrieval and difficulty in discourse organization. We did not find selective impairments on comprehension, denomination, repetition, reading, and writing. Laboratory tests were in range except for a slight increase in ammonia and amylase (non-specific findings); slight hypovitaminosis D and subclinical hyperthyroidism (with normal autoimmune screening with anti-TPO, anti-thyroglobulin, anti-TRAK). No further abnormalities were present in electrolytes, glucose, PCR, VES, sidero-vitamin levels (including B1 e B12). The autoimmune and infectious screening was negative.

**Table 1:** Neuropsychology tests panel.

Domain of function	Task	Raw score	Adjusted score	Cutoff	Equivalent score
Global cognitive functioning	Mini-Mental State Examination (MMSE)	23/30	21	24
Language	Boston Naming Test	13/15	12	11	2
	Phonemic fluency	15	17.50	17.35	1
	Semantic fluency	25	27	25	1
Attention and processing speed	Trail Making Test, Part A (seconds)	42	25	93	4
	Trail Making Test, Part B (seconds)	313	255	282	1
	Trail Making test, B-A (seconds)	271	230	186	0
Memory	Digit Span forward	4/9	4.13	4.26	0
	Digit span backward	3/8	3.19	2.65	1
	Corsi span forward	3/9	3.15	3.46	0
	Corsi span backward	0/8	-	3.08	0
	Story recall test	3/28	2.50	8.00	0
	Rey-Osterrieth Complex figure test (ROCF)—Recall	8.5/36	12	9.47	2
Executive function	Frontal Assessment battery (FAB)	10/18	10.34	11.60	0
Visuocostructional skills	Rey-Osterrieth Complex figure test (ROCF)—Copy	36/36	-	28.88	4

*Performance on neuropsychological testing presented as raw scores, adjusted scores (for age and education) and equivalent scores. Equivalent score (Capitani and Laiacona, 1997) is a 5-point scale where neuropsychological scores are standardized after adjustment for age and education. ES = 0 indicates a pathological performance. ES = 1 a borderline performance. ES ≥ 2 a normal performance. Neuropsychological assessment results are organized by domain of function*.

**Table 2:** Neuropsychological exam for aphasia (ENPA).

Task	Sub-task	Raw Score	Adjusted score	Cut-off	Interpretation
Repetition	Words	10/10	–	8.8	-
	Not-words	5/5	–	2.0	-
	Phrases	3/3	–	3.0	-
Reading	Words	10/10	–	6.4	-
	Not-words	5/5	–	4.0	-
	Phrases	2/2	–	1.3	-
Writing	Words	8/10	7.4	6.3	-
	Not-words	3/5	2.3	1.4	-
	Phrases	2/2	–	0.6	-
Denomination	Nouns/oral	10/10	–	8.2	-
	Nouns/writing	5/5	–	2.7	-
	Verbs/oral	8/10	7.5	6.1	-
	Verbs/writing	3/5	2.6	3.0	-
	Colors/oral	5/5	–	4.0	-
Comprehension	Words/hearing	20/20	–	18.4	-
	Words/visual	19/20	18.8	17.0	-
	Phrases/hearing	12/14	11.9	11.6	-
	Phrases/visual	12/14	11.6	11.3	-
Words generation	Letter F	5	4.1	5.7	*
	Letter A	6	5.1	4.8	-
	Letter S	2	1.1	5.8	*
	Animals	10	9.2	10.3	*
	Objects	11	8.1	8.5	*
Spoken picture description		–	–	–	Frequent pauses for word-finding, impaired connecting speech, a problem with discourse organization^*^

Performance on language testing presented as raw scores, adjusted scores (for age and education) and interpretation of the results. – Indicates a normal performance; ^*^Indicates a pathological performance.

**Figure 1:**
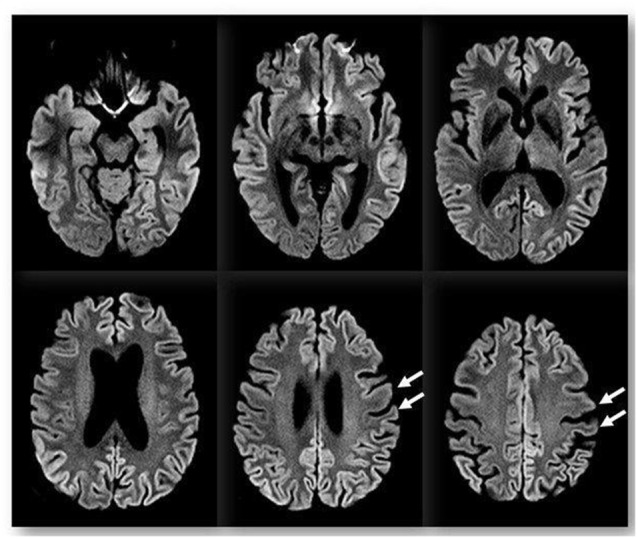
Brain magnetic resonance imaging (MRI). Diffusion-weighted imaging (DWI) with *b* value of 1,000 s/mm^2^ showing bilateral and diffuse supratentorial cortical diffusion signal changes with a restriction on ADC map (not shown) and slight left lateralization. The peri-rolandic cortex is spared (arrows). There is no involvement of subcortical structures.

**Figure 2:**
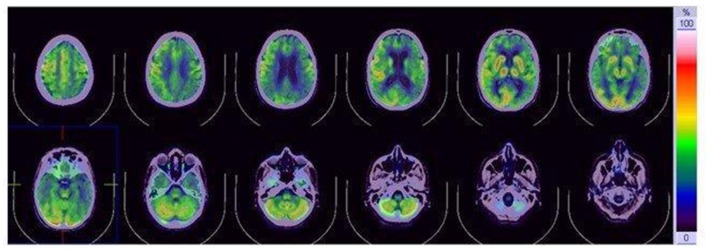
[^18^F]fluoro-2-deoxy-D-glucose positron emission tomography (FDG-PET). FDG-PET reduced glucose metabolism in the temporo-parietal and frontal lobe, with slight left lateralizatio. No involvement of subcortical structures is present.

We performed an extensive panel to search for paraneoplastic antibodies with negative results. Moreover, chest and abdomen CT scan with iodinated contrast were unremarkable. CSF examination was in range. CSF protein 14–3–3 was negative. The dosage of protein TAU and phospho-TAU was normal (TAU 284 ng/L—ref. <360 ng/L; p-TAU 30 ng/L—REF <60 ng/L), while Aβ42 was slightly reduced (Aβ42 367 ng/L—ref.>, 450 ng/L). Electroencephalogram (EEG) at baseline showed non-specific findings, with intermittent slight slow-waves abnormalities, mostly in the left frontal, central and temporal regions. Brain magnetic resonance imaging (MRI) showed bilateral and diffuse supratentorial cortical diffusion restriction, more evident in the parietal lobes and posterior cingulum, with sparing of the peri-rolandic cortex. No clear cortical signal abnormalities were visible on T2 weighted or T2-fluid-attenuation-recovery (FLAIR) images and no enhancement was visible after contrast media administration. Brain MRI abnormalities were visible since the first evaluation. Basal ganglia and thalami were never involved (Figure 1). Abnormalities remained stable at 2 and 6 weeks follow up MRI exams (not shown). FDG-PET ([^18^F]-fluoro-2-deoxy-D-glucose positron emission tomography) showed left lateralized reduced glucose metabolism in temporal, parietal and frontal lobes, partially overlapping with the cortical diffusivity abnormalities seen on MRI. No involvement of subcortical structures was present (Figure 2). MRI findings were strongly indicative of CJD. However clinical, laboratory and EEG findings were not sufficient to fulfill the diagnostic criteria of CJD which applied at the time of clinical assessment (Vitali et al., 2011). Indeed, neither myoclonus, visual or cerebellar signs or akinetic mutism were present; periodic sharp wave complexes were not visible at baseline EEG and the CSF assay for 14–3–3 was negative. We, therefore, performed a real-time quaking-induced conversion (RT-QuIC) assay on a frozen sample of corticospinal fluid (CSF), which showed a positive result. Notably, CSF was clear, protein and white blood cell levels in CSF were in range, respectively 271 mg/L (range 200–400) and 0,7/μl (normal value <5/μl). In the following months, the patient experienced a rapid worsening of symptoms, with symptomatic epilepsy. A detailed neuropsychological follow-up could not be repeated. Neither myoclonus, tremors or involuntary movements were present during the course of the disease. Notably, a long-term EEG monitoring, performed in February 2017, showed most evocative findings, with single triphasic waves in both waking and sleeping status compatible with mild encephalopathy and slight phasic periodism during the night. The patient died in May 2018 after the recurrence of an epileptic seizure. She developed pneumonia probably due to bronchoaspiration with consequent respiratory failure. Pathology proved the diagnosis of sporadic CJD, subtype MM/MV 2C.

## Discussion

This is a case of RPD induced by CJD which exhibited unusual clinical and EEG manifestations, yet typical MRI findings, which may be relevant to physicians to consider the CJD etiology in similar clinical pictures. RPD refers to a condition characterized by a quick decline in more than one cognitive domain with functional disability in a short time (Geschwind, 2016). The differential diagnosis of RPD includes many treatable conditions that our patient multimodal work-up allowed to rule out. Among these, paraneoplastic autoimmune encephalopathies, infections, toxic or metabolic conditions, neoplasms, and other conditions such as dural arteriovenous fistula (DAVF) that sometimes manifest as RPD (Geschwind, 2016).

In the differential diagnosis, we considered an atypical presentation of primary progressive aphasia (PPA) since language skills were involved from the beginning of the disease. The clinical profile of PPA is a gradually progressive deficit in language function with relative sparing of other intellectual functions (Mesulam, 2003; Gorno-Tempini et al., 2011). Among the different forms of PPA, the cognitive profile of our patient resembled more that found in logopenic progressive aphasia (LPA). LPA is characterized by word-finding difficulty with frequent pauses in spontaneous speech, impaired single word retrieval and naming, difficulty in repeating sentences, phonological errors and confrontation naming. Comprehension, motor speech and word repetition are spared. The language profile of our patient was not fulfilling the criteria for the other forms of PPA: in the non-fluent agrammatic aphasia and semantic aphasia. The first condition is characterized by disturbances of language production, distorted articulation (apraxia of speech), syntactic simplification with telegraphic language, without a deficit in word comprehension. The second condition, also named semantic PPA, is characterized by severe anomia, impaired word comprehension with loss of meaning of the words, gradual disintegration of semantic knowledge about people and objects, with preservation of the phonological and syntactic aspects of language (Gorno-Tempini et al., 2011). Consistent with our language assessment, the patient could repeat word, not-words and sentences, but produced a reduced output, clearly showed by impaired performance on fluency tasks and spoken picture description, demonstrating an executive derangement in language tests as well as in other executive tasks (Trail Making Test, Part B, digit span backward). This is in line with a recent report that outlined that language disorders in prion diseases represent a dynamic aphasia in the context of a prominent dysexecutive syndrome (Caine et al., 2018). PET findings could be compatible with LPA even though hypometabolism was found to extend beyond the temporoparietal areas. However, the rapid evolution of global cognitive impairment was not supporting this hypothesis.

The possibility of an Early-onset Alzheimer’s disease (EOAD) was also considered. EOAD manifests in younger subjects <65 years and is characterized by impairment in language, visuospatial and behavioral executive domains with sparing of episodic memory, which is typically involved in late-onset Alzheimer’s disease (LOAD; Palasí et al., 2015; Mendez, 2019). EOAD patients have a more aggressive clinical course (Koedam et al., 2008; Stanley and Walker, 2014), and age-related psychosocial needs (Rosness et al., 2010). Decreased CSF level of Ab42 could support the hypothesis of Alzheimer’s disease however tau and phospho-tau were not elevated. Also, FDG_PET uptake was decreased beyond the temporoparietal cortex.

In light of the characteristic of MRI findings, the diagnosis of CJD was considered. CJD, although rare, is the most common human prion diseases, a group of lethal transmissible neurodegenerative diseases, related to the conversion of a normal cellular protein, the prion protein (PrPC), into a misfolded form (PrPSc) which accumulates in neuronal cells leading to intracellular spongiform changes and neuronal loss (Prusiner, 1998). Human prion diseases occur in sporadic, genetic and acquired forms. Sporadic CJD (sCJD) is the most common form and is related to the spontaneous conformational conversion of PrP^C^. Genetic forms, caused by mutations in the gene PRNP encoding for PrP^C^, include familial CJD (fCJD), Gerstmann-Sträussler-Scheinker syndrome (GSS) and fatal familial insomnia (FFI). The acquired forms, resulting from the human-to-human transmission, include kuru and iatrogenic CJD (iCJD). Kuru, related to cannibalism, is now considered to be extinct. ICJD has been reported to occur by transmission *via* contaminated neurosurgical instruments such as intracerebral EEG needles, human dura mater grafts, inoculation with human pituitary hormones or corneal transplantation. A further iatrogenic form, variant Creutzfeldt–Jakob disease (vCJD), results from bovine to human transmission of the agent of bovine spongiform encephalopathy (BSE); a human-to-human transmission may also occur.

According to the molecular classification proposed by *Parchi e Gambetti* (Parchi et al., 2012), different molecular subtype of sCJD are distinguished based on the PRNP gene codon 129 genotype, homozygous or heterozygous for methionine (M) or valine (V), and the pathologic prion protein (PrP^Sc^) type, that is classified as type 1 or type 2 depending on the size and electrophoretic mobility at Western blot of the protease-resistant core fragment (PrPres). Six phenotypes are therefore distinguished: MM1, MM2, MV1, MV2, VV1, or VV2. MM2 can be further divided into two subgroups based on histopathological criteria: MM2C, cortical form, with predominant cortical pathology and MM2T, thalamic form, with typical atrophy of thalamic and inferior olivary nuclei. The MV2 group has also been divided into two distinct subtypes based on pathological criteria MV2C (MV2 cortical type) and MV2K (MV2 kuru plaque-type). Since both MM1/MV1 and MM2C/MV2C share the same clinicopathological features, they have been merged into single entities, MM/MV1 subtype and the MM/MV2C subtype. In conclusion, six different subtypes are distinguished in the current classification of sCJD: MM/MV1, MM/MV2C, MM2T, MV2K, VV1 or VV2 (Parchi et al., 2012).

CJD brain MRI abnormalities typically involve the cortex and the basal ganglia (putamen and caudate), with diffusion restriction variably associated with T2 and FLAIR hyperintensity. Cortical involvement may be focal or diffuse, symmetric or asymmetric; the perirolandic cortex is usually spared. Thalamic involvement (unilateral or bilateral) has been described in cases of variant-CJD but can be found in sporadic forms as well. Thalamic signal abnormalities are known as MRI “hockey stick sign,” since they are located in the pulvinar and dorsomedial thalamic nuclei, resembling the shape of a hockey stick. Diffusion restriction in the cerebellum has been reported in atypical cases (Fragoso et al., 2017). Similar MRI abnormalities may be found in hypoxic/anoxic brain injury, encephalitis, metabolic conditions such as hepatic encephalopathy or hypoglycaemic encephalopathy, and status epileptics, therefore MRI finding must be interpreted in the appropriate clinical setting.

To date, a diagnosis of “definite CJD” can be made just upon neuropathologic examination, with prion protein identification by immunochemistry or Western blotting. An *in vivo* diagnosis of “probable CJD” has been previously based on the WHO diagnostic criteria established in 1998 (WHO, 1998), with the following revisions in 2009 by the European MRI-CJD Consortium (Zerr et al., 2009) and in 2011 by the University of California, San Francisco (Vitali et al., 2011). According to WHO revised criteria a diagnosis of possible CJD was possible in the presence of progressive dementia plus two other clinical findings among myoclonus, pyramidal/extrapyramidal symptoms, visual/cerebellar dysfunction, and akinetic mutism, plus EEG evidence of periodic sharp wave complexes (PSWCs) or elevated CSF 14.3.3 protein or typical MRI findings, such as high-signal intensity on either FLAIR or DWI in both the putamen and the caudate nucleus or at least two cerebral cortical regions, from either the temporal, occipital, or parietal cortices, not including frontal or limbic regions. The National CJD Research and Surveillance Unit (NCJDRSU) in 2017 (Unit NCR and S PROTOCOL, 2017) and the Center for Disease Control and Prevention (CDC) in 2018 (Diagnostic-Criteria, 2018) have introduced new diagnostic criteria incorporating the use of a novel ultrasensitive seeding assay, the real-time quaking-induced conversion assay (RT-QuIC). This assay can detect the amplified pathological prion protein in the CSF or the olfactory mucosa with very high sensitivity and specificity (Bongianni et al., 2017; Foutz et al., 2017). The assay is based on the ability of the misfolded pathological prion protein (PrPSc) to induce conversion of the normal prion protein (PrP) to the misfolded form, with subsequent protein aggregation. The formation of aggregates can be monitored in real-time using a fluorescent dye. These new criteria now allow a diagnosis of “probable CJD” just in the presence of progressive neuropsychiatric disorder and positive RT-QuIC in CSF or other tissues, extending the diagnosis to cases with atypical clinical presentation and non-supportive EEG and/or MRI findings. Notably, EEG PSWCs are found in approximately 73% of patients with sCJD, usually in the late stage of the disease while MRI abnormalities are found in about 83% of patients. Protein 14–3–3 has been reported to have a good sensitivity for CJD (up to 85–95%), however, it is not specific (Zerr et al., 2009).

The interpretation of the RT-QuIC assay is affected by the presence of raised red and white cells counts and elevated total protein concentrations in the CSF. Red cells in CSF samples inhibit the RT-QuIC response, with fewer replicates and longer reaction times. A cut-off of <1,250 × 10^6/L^ red blood cells is recommended. High CSF total protein concentrations of >1.0 g/L and raised white blood cells may result in an RT-QuIC response with a high and fluctuating baseline that can be misinterpreted as a positive RT-QuIC result. CSF samples for RT-QuIC analysis are required to be clear, with a white cell count of <10 × 10^6/L^ and a total protein concentration of <1 g/L. CSF RT-QuIC is not affected if CSF samples are stored at room temperature or 4°C for up to 8 days and is not affected by repeated freeze and thaw cycles. RT-QuIC can be run on CSF samples but also olfactory neuroepithelium obtained by nasal brushing (Green, 2019). RT-QuIC has demonstrated high diagnostic value but has limited prognostic value since different sCJD subtypes generate very similar RT-QuIC reaction products (Piconi et al., 2019).

Other biochemical analysis methods have been developed for the diagnosis of CJD. Among these, Sodium Phospho-Tungstic Acid (NaPTA) precipitation/western blotting and Conformation Dependent Immunoassay (CDI) that, so far, have not been tested for use in routine diagnostics or screening, and Protein Misfolded cyclic amplification (PMCA) that is less sensitive for sCJD PrPSc (Franceschini et al., 2017).

The role of positron emission tomography (PET) in CJD is not well elucidated compared to other neurodegenerative diseases. A left-lateralized frontal and parietal hypometabolism, as we found in our patient, has been shown in a cohort of CJD patients (Renard et al., 2017). PET abnormalities do not have a strict anatomical correlation with MRI abnormalities and may precede them. Basal ganglia hypometabolism is not commonly detected (Mente et al., 2017).

In conclusion, in our case, the complementary examinations carried out, and in particular, the RT-QuIC test in CSF, was fundamental to make the correct diagnosis and thus differentiate between the possible etiologies of RPD.

## Data Availability Statement

All datasets generated for this study are included in the article.

## Ethics Statement

Diagnostic work-up and case report descriptions were conducted according to the principles expressed in the Declaration of Helsinki, the institutional regulation and Swiss laws and guidelines. Written informed consent for the publication of the content of this case report was obtained from the patient’s daughter after her death.

## Author Contributions

EPro, SR, and IB wrote the manuscript, made table and figures, reviewed the literature. EPro interpreted brain MRI results. LS performed neurological evaluations of the patient. SR reviewed the neuropsychological and language evaluations of the patient. LS and EPra performed the final manuscript review.

## Conflict of Interest

The authors declare that the research was conducted in the absence of any commercial or financial relationships that could be construed as a potential conflict of interest.
